# Assessment of immunotherapy response in intracranial malignancy using semi-automatic segmentation on magnetic resonance images

**DOI:** 10.3389/fimmu.2022.1029656

**Published:** 2022-12-14

**Authors:** Jia Tan, Chang Liu, Yan Li, Yiqi Ma, Ruoxi Xie, Zheng Li, Hengjiang Wan, Su Lui, Min Wu

**Affiliations:** ^1^ Huaxi MR Research Center, Department of Radiology, Functional and Molecular Imaging Key Laboratory of Sichuan Province, West China Hospital, Sichuan University, Chengdu, China; ^2^ Department of Radiology, West China Hospital, Sichuan University, Chengdu, China

**Keywords:** immunotherapy response, RECIST1.1, intracranial malignancy, magnetic resonance imaging, semi-automatic segmentation

## Abstract

**Objective:**

To explore multi-aspect radiologic assessment of immunotherapy response in intracranial malignancies based on a semi-automatic segmentation technique, and to explore volumetric thresholds with good performance according to RECIST 1.1 thresholds.

**Methods:**

Patients diagnosed with intracranial malignancies and treated with immunotherapy were included retrospectively. In all MR images, target lesions were measured using a semi-automatic segmentation technique that could intelligently generate visual diagrams including RECIST 1.1, total volume, and max. 3D diameter. The changes in parameters were calculated for each patient after immunotherapy. The ROC curve was used to analyze the sensitivity and specificity of the size change of the legion. This was useful to find new volumetric thresholds with better efficiency in response assessment. The changes in total volume were assessed by conventional volumetric thresholds, while RECIST 1.1 thresholds were for the max. 3D diameter. A chi-square test was used to compare the concordance and diagnostic correlation between the response assessment results of the three criteria.

**Results:**

A total of 20 cases (average age, 58 years; range, 23 to 84 years) and 58 follow-up MR examinations after immunotherapy were included in the analysis. The P-value of the chi-square test between RECIST 1.1 and total volume is 0 (P <0.05), same as that in RECIST 1.1 and max. 3D diameter. The kappa value of the former two was 0.775, and the kappa value for the latter two was 0.742. The above results indicate a significant correlation and good concordance for all three criteria. In addition, we also found that the volumetric assessment had the best sensitivity and specificity for the immunotherapy response in intracranial malignancies, with a PR threshold of −64.9% and a PD threshold of 21.4%.

**Conclusions:**

Radiologic assessment of immunotherapy response in intracranial malignancy can be performed by multiple criteria based on semi-automatic segmentation technique on MR images, such as total volume, max. 3D diameter and RECIST 1.1. In addition, new volumetric thresholds with good sensitivity and specificity were found by volumetric assessment.

## Introduction

Intracranial malignancies, including primary and metastatic tumors, seriously endanger human health. In recent years, in addition to surgery, radiotherapy, and chemotherapy, immunotherapy has achieved exciting progress in the treatment of intracranial malignancies (1). According to iRANO (immunotherapy Response Assessment for Neuro-Oncology) criteria, the assessment process of immunotherapy response in patients with neuro-oncological malignancies mainly involves two aspects: one is the assessment of radiological progression on follow-up oncologic imaging; the other is the assessment of new or substantially worsening neurological deficits indicating clinical decline unrelated to a comorbid event or concurrent medication. Both aspects together determine whether the neurosurgeon should change the clinical treatment decision for the patient (2, 3).

From the point of view of MR imaging, accurate radiological assessment of the size changes in intracranial malignancies after immunotherapy is still very important, which can help reflect the prognosis of patients more precisely and make better clinical treatment decisions. iRANO criteria guidelines stated that WHO (WHO = ∑ (long diameter ∗ short diameters) of target lesions) criteria were commonly used for radiological assessment of malignant gliomas, which had the same evaluation efficiency with RECIST 1.1 (4). For brain metastases, according to the RANO-BM (Response Assessment in Neuro-Oncology Brain Metastases) criteria, RECIST 1.1 (RECIST = ∑ (longest diameter of target tumors, shortest diameter of target lymph nodes)) criteria (5, 6) were used for radiological assessment. Although RECIST 1.1 is the most widely used criteria, it still has some shortcomings. Calculated values of RECIST 1.1 only represents the change in the longest axial diameter, but not the changes in all directions, so the authentic tumor size may be underestimated or overestimated, let alone the difficulty of accurate measurement of the tumor sizes with irregular shapes (7).

At present, a better way to solve the difficulties in RECIST 1.1 is volumetric assessment. Previous studies on size measurement of Vestibular Schwannomas (VS) found that linear measurements underestimated growth rate and were not as sensitive as volumetric measurements to tumor size changes (8). Meanwhile, an earlier study of brain metastases showed that the semi-automated segmentation technique based on CE-MRI (contrast-enhanced *T*_1_-weighted magnetic resonance imaging) showed lower intra-observer and inter-observer variability in volumetric measurement compared with unidimensional measurements, which could better reflect the real tumor size (7). Similarly, the potential applications of volumetric measurement have also been widely validated in other diseases, such as intrahepatic malignancies, lung metastases, rectal cancers, etc. (9–14).

Up until now, the main problem with volumetric assessment is the lack of a standardized threshold, which hinders the accurate assessment of remission and progress (10, 14). Because in some previous studies, the volumetric thresholds were derived from the RECIST 1.1 thresholds using a mathematically theoretical spherical formula (15). Therefore, in this study, we tried to find new volumetric thresholds for better diagnostic performance using the available data. Volumetric measurement was previously considered to be time-consuming and laborious. However, with the development of semi-automatic segmentation techniques, volumetric measurement may become more intelligent, efficient, and accurate.

Based on the existing semi-automatic segmentation technique, we attempt to find multiple radiologic criteria for immunotherapy response assessment in intracranial malignancies by analyzing CE-MRI images (16–18). Meanwhile, we will also seek new volumetric thresholds corresponding to the established RECIST 1.1 thresholds to assess the immunotherapy response of intracranial malignancies more sensitively and specifically.

## Materials and methods

### Screening of cases

We retrospectively scanned and analyzed the cases of patients diagnosed with intracranial malignancies and treated with immunotherapy from August 2018 to June 2022. The patients had primary gliomas or brain metastases from various malignancies, including lung adenocarcinoma, small cell lung cancer, and malignant melanoma, and follow-up MR (magnetic resonance) examinations were performed before and after immunotherapy. The inclusion criteria included the following:

1. At least one measurable lesion was present on the MR images (6).
2. The MR images had no artifacts that may affect the observation of target lesions, including motion artifacts, susceptibility artifacts, metal artifacts, etc.
3. The slice thickness of each follow-up CE-MRI images should be 1 mm.
4. Immunotherapy should be continued between the two follow-up MR examinations.

**Figure 1:**
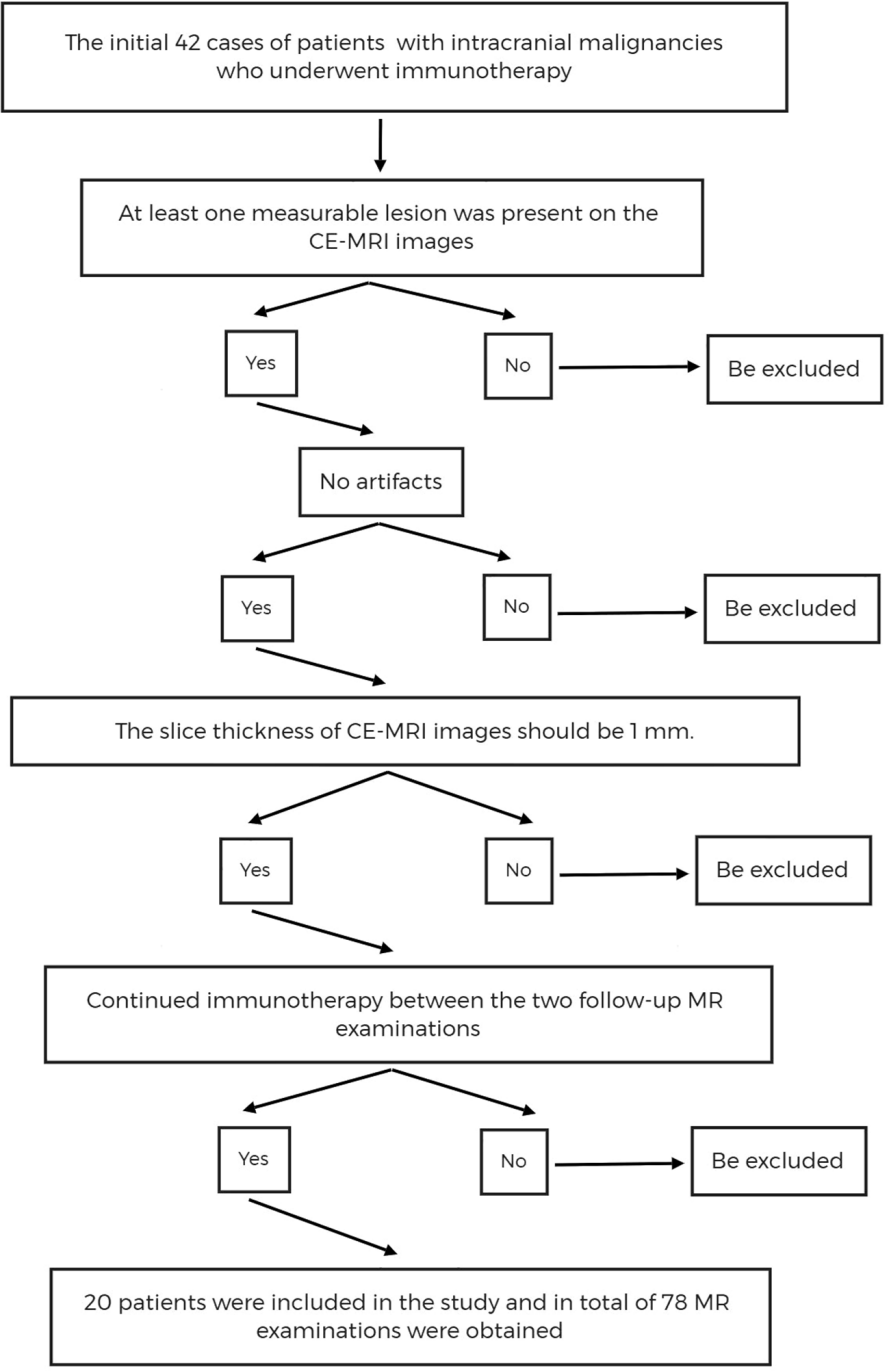
The flow chart of cases screening. CE-MRI, Contrast Enhanced T1-weighted Magnetic Resonance Imaging.

The flow chart for case screening is shown in **Figure 1**.

### MRI acquisition

All the MR images were acquired randomly on 1.5 Tesla or 3.0 Tesla imaging systems from different scanner manufacturers, including Siemens, GE (General Electrical), Philips, and United Imaging. The critical parameters of CE-MRI protocols are shown in **Table 1**. The GBCAs (Gadolinium-based Contrast Agents) were used for CE-MRI, and the specific contrast agent information is as follows: gadoteridol injection (BIPSO GmbH), gadopentetate dimeglumine injection (Beijing Beilu Pharmaceutical Co., Ltd.), gadobutrol injection (Bayer Vital GmbH), and gadoteric acid meglumine salt injection (Jiangsu Hengrui Pharmaceutical Co., Ltd.). The dose of contrast agent was 0.1 mmol/kg.

**Table 1:** The critical parameters of CE-MRI protocols.

Parameters	Siemens	GE	Philips	United Image
Sequence	3D MPRAGE	3D FSPGR	3D TFE	3D GRE_fsp
Field of view (cm)	25	24	23	23
Acquisition matrix (pixels)	256 × 256	256 × 256	232 × 230	232 × 230
Thickness (mm)	1	1	1	1
Slice gap (mm)	0	0	0	0
Repetition time (ms)	1,730	8.2	Shortest	15.1
Echo time (ms)	2.26	2.6	Shortest	6.2
Number of excitations	1	1	1	1
Flip angle (degree)	9	12	8	10
Bandwidth (Hz)	130	62.5	270.7	200
Orientation	Sagittal	Axial	Sagittal	Sagittal

3D MPRAGE, Three-dimensional Magnetization Prepared Rapid Gradient Echo; 3D FSPGR, Three-dimensional Fast Spoiled Gradient-echo; 3D TFE, Three-dimensional Turbo Field Echo; 3D GRE fsp, Three-dimensional Gradient Recalled Echo fast spoil.

### Data analysis

All filtered MR data sets were loaded into the post-processing workstation, the ISP (Intellispace Philips Portal) and target lesions were semi-automatically delineated and analyzed using Multimodal Tumor Tracking (MMTT) software. MMTT software is a comprehensive technique based on regional growth and morphological image processing algorithms.

All routine follow-up MR images were reviewed by the senior radiologists (CL and YL) to qualitatively assess the presence of intracranial malignancy. Since previous studies had shown excellent intra-observer and inter-observer consistency with the semi-automated segmentation technique (7). Therefore, just an experienced radiologist independently measured, and all the target lesions were independently analyzed by the radiologist, but after discussing and reaching an agreement by two readers, some borderline cases were reviewed with two radiologists for consensus.

**Figure 2:**
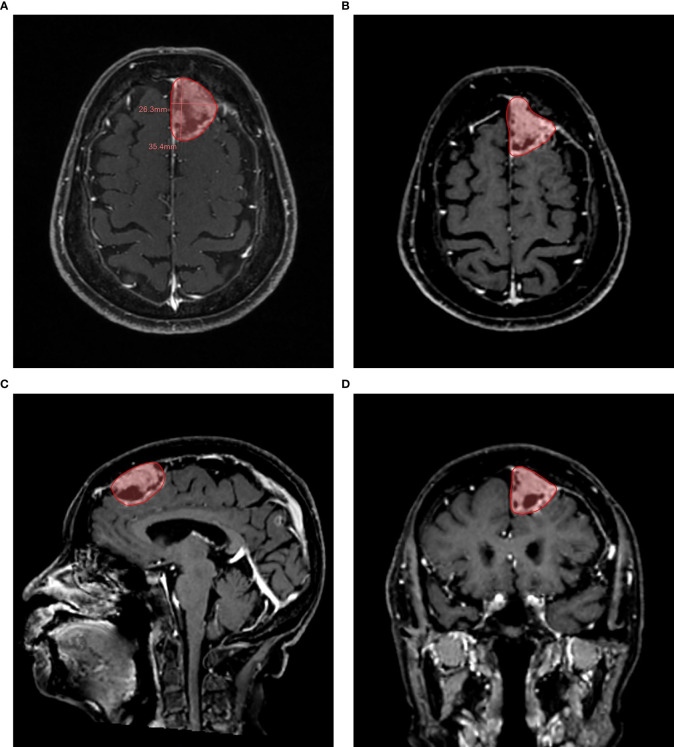
Example of a semi-automatic segmentation. The follow-up 3D CE-MRI of an 84-year-old female patient with brain metastases from lung adenocarcinoma. **(A)**: Automatic measurement of maximum long diameter and maximum axial diameter. **(B)**: Semi-automatic segmentation in axial plane. **(C)**: Semi-automatic segmentation in sagittal plane. **(D)**: Semi-automatic segmentation in coronal plane.

The number of target lesions was confirmed according to the RECIST 1.1 criteria, which required one lesion at least and five lesions at most. When the number of lesions is greater than five, only large and well-defined lesions should be selected. The target lesions included round-like lesions and heterotypic lesions. The delineation boundary was defined by the disruption area of the blood–brain barrier. Automatic segmentation could be started by clicking the mouse in the center of the enhanced area in axial view, and it could be adjusted manually if necessary. When delineating the target lesion, the boundary was identified in as many directions as possible (**Figure 2**). In the segmentation process, each time spent was measured in seconds. After that, multidimensional information could be obtained within a few seconds, including the detailed values of RECIST 1.1, total volume, max. 3D diameter (maximum three-dimensional diameter), etc.

Taking the assessment results of target lesions before immunotherapy as a baseline, the changes in all results, including RECIST 1.1, total volume, and max. 3D diameter, were calculated for all patients at follow-up MR examinations after immunotherapy (7). Here, size changes of target lesions are calculated as the ratio of the difference between follow-up value and baseline value to the baseline value.

Radiographic response assessment for the change in max. 3D diameter was assessed based on RECIST 1.1 criteria, and radiographic response assessment for total volume was performed according to the theoretical volume thresholds. The detailed response thresholds are shown in **Table 2**.

**Table 2:** Summary of the response criteria.

Response assessment	Sum of size change in tumors’ Size		
	Total volume	RECIST1.1	Max. 3D diameter
CR (Complete response)	none	none	none
PR (Partial response)	≤−65%	≤−30%	≤−30%
SD (Stable disease)	>−65%, <73%	>−30%, <20%	>−30%, <20%
PD (Progressive disease)	≥73%	≥20%	≥20%
References	(10, 19, 20)	(5)	

### Statistical analysis

A total of three groups of data were collected and analyzed, including changes in RECIST 1.1 data, total volume data, and max. 3D diameter data. After setting the PR (partial response) threshold to −30% and the PD (progressive disease) threshold to 20%, PR and PD were taken as the state variables. Differences in immunotherapy response between the three groups were compared pairwise using the chi-square test (21), and the null hypothesis called H0 was set to “the three groups of data were not correlated.” Both the Fisher exact text values and corresponding p-values were recorded. If P <0.05, it indicated that there was statistical significance among the three groups; if P >0.05, it indicated that there was no statistical significance among the three groups. Meanwhile, the Kappa value of each test was recorded to observe the concordance of the immunotherapy response among total volume, max. 3D diameter, and RECIST 1.1. Kappa value ≥0.75, the concordance was good; 0.75≥ Kappa value ≥0.4, the concordance was general; Kappa value <0.4 showed poor concordance.

Size changes of total volume and max. 3D diameter were taken as the test variables to draw the ROC curve (receiver operator characteristic curve) (22). Then, the size change corresponding to the maximum sum of sensitivity and specificity was selected as the optimal threshold. The AUC (area under the curve) value was also recorded at the same time. If AUC value = 1, the assessment effectiveness was perfect; if 0.85 <AUC value >0.95, the assessment effectiveness was very good; if 0.85 <AUC value >0.95, the assessment effectiveness was general; if AUC value <0.5, the assessment effectiveness was poor.

Finally, RECIST 1.1 was used as the “gold standard,” and cases were selected as examples to further analyze the response assessment between total volume, max. 3D diameter, and RECIST 1.1. All statistical analyses were performed using statistical software IBM SPSS Statistics 26.

## Results

### Patient data

After initial screening, there were a total of 42 cases of patients with intracranial malignancies who underwent immunotherapy. After further screening using inclusion criteria, 20 patients (males: 15; females: 5; average age: 58 years; range: 23–84 years) were included in this study. A total of 78 MRI scans obtained from these patients between September 2018 and June 2022 were used for analysis. Excluding the 20 MR scans performed before immunotherapy, the final number of MR scans used to calculate the size change rates was 58. There were three discrepant cases, all of which were derived from glioma patients, which may be due to the large boundary atypia of gliomas. Clinical information and follow-up MRI information for these cases are shown in **Table 3**.

**Table 3:** Detailed information of measurements.

Intracranial Malignancy	Clinical diagnosis	Number of cases	Age	Gender	Immunotherapy drugs	Number of MR examinations
Glioma	Glioblastoma (WHO IV)recurrence after surgery	3	58	M	Pembrolizumab	4
	Glioblastoma (WHO IV)recurrence after surgery		45	F	Toripalimab and Bevacizumab	4
	Glioblastoma (WHO IV)recurrence after surgery		53	M	Pembrolizumab	2
	Oligodendroglioma (WHO III)recurrence after surgery	1	22	M	Camrelizumab	4
	Oligodendroglioma (WHO II)recurrence after surgery	1	44	M	Camrelizumab and Bevacizumab	6
Brain metastasis	Originated from lung adenocarcinoma	11	71	M	Pembrolizumab	2
	Originated from lung adenocarcinoma		63	M	Tislelizumab	3
	Originated from lung adenocarcinoma		50	M	Camrelizumab and Bevacizumab	4
	Originated from lung adenocarcinoma		55	M	Sugemalimab	3
	Originated from lung adenocarcinoma		54	M	Toripalimab	2
	Originated from lung adenocarcinoma		66	M	Bevacizumab and Tislelizumab	2
	Originated from lung adenocarcinoma		80	F	Sintilimab and Bevacizumab	2
	Originated from lung adenocarcinoma		65	M	Pembrolizumab	4
	Originated from lung adenocarcinoma		83	F	Sintilimab and Bevacizumab	13
	Originated from lung adenocarcinoma		M	53	Pembrolizumab and Bevacizumab	7
	Originated from lung adenocarcinoma		63	M	Pembrolizumab	2
	Originated from small cell lung cancer	2	53	M	Atezolizumab and Tislelizumab	2
	Originated from small cell lung cancer		74	M	Atezolizumab and Tislelizumab	2
	Originated from malignant melanoma of the rectum	1	53	F	Toripalimab	7
	Originated from malignant melanoma of the left groin	1	63	F	Pembrolizumab	3

Detailed information about the values and MR examinations are shown in **Table 4**.

**Table 4:** Detailed information of measurements.

Total Patients	20
Total number of MR examinations	78
Total number of MR examinations after immunotherapy	58
Total Number of target lesions	156
Examinations per patient (mean ± SD)	3.9 ± 2.67
Target lesions each examination (mean ± SD)	2 ± 1.31
Generated values of RECIST1.1 each time (mean ± SD)	39.38 ± 28.91
Generated values of total volume each time (mean ± SD)	15.52 ± 23.34
Generated values in sum of max. 3D diameter each time (mean ± SD)	44.26 ± 31.14
Size change of RECIST 1.1 (×100%)	0.39 ± 1.34
Size change of total volume (×100%)	1.44 ± 4.58
Size change of max. 3D diameter (×100%)	0.43 ± 1.43
Measurement time (s)	428.83 ± 88.47

### Correlation and concordance

Detailed comparison results of immunotherapy response assessment between RECIST 1.1 and total volume by chi-square test are shown in **Table 5**. The Fisher exact test value was 59.70, and its corresponding P-value was zero, which was less than 0.05, indicating that there was statistically significance. The above results for total volume showed a strong correlation with RECIST 1.1. Similarly, **Table 6** showed the assessment results between RECIST 1.1 and max. 3D diameter. While Fisher’s exact value was 57.382, its corresponding P-value was also zero. Besides, the Kappa value was higher than 0.75. There was high concordance between RECIST 1.1 and max. 3D diameter.

**Table 5:** Comparisons of response assessment between RECIST 1.1 and total volume.

		RECIST 1.1			
		PD	PR	SD	Total
	PD	14	0	7	21
Total volume	PR	0	9	0	9
	SD	2	0	26	28
	Total	16	9	33	58

Fisher exact test value: 59.70; Exact sig (2-sided): 0.000; Kappa value: 0.742.

**Table 6:** Comparisons of response assessment between RECIST 1.1 and max. 3D diameter.

		RECIST1.1			
		PD	PR	SD	Total
	PD	19	1	2	22
Max. 3D diameter	PR	0	7	2	9
	SD	2	1	24	27
	Total	21	9	28	58

Fisher exact test value: 57.382; Exact sig (2-sided): 0.000; Kappa value: 0.775.

### ROC curve and new volumetric thresholds

**Figure 3:**
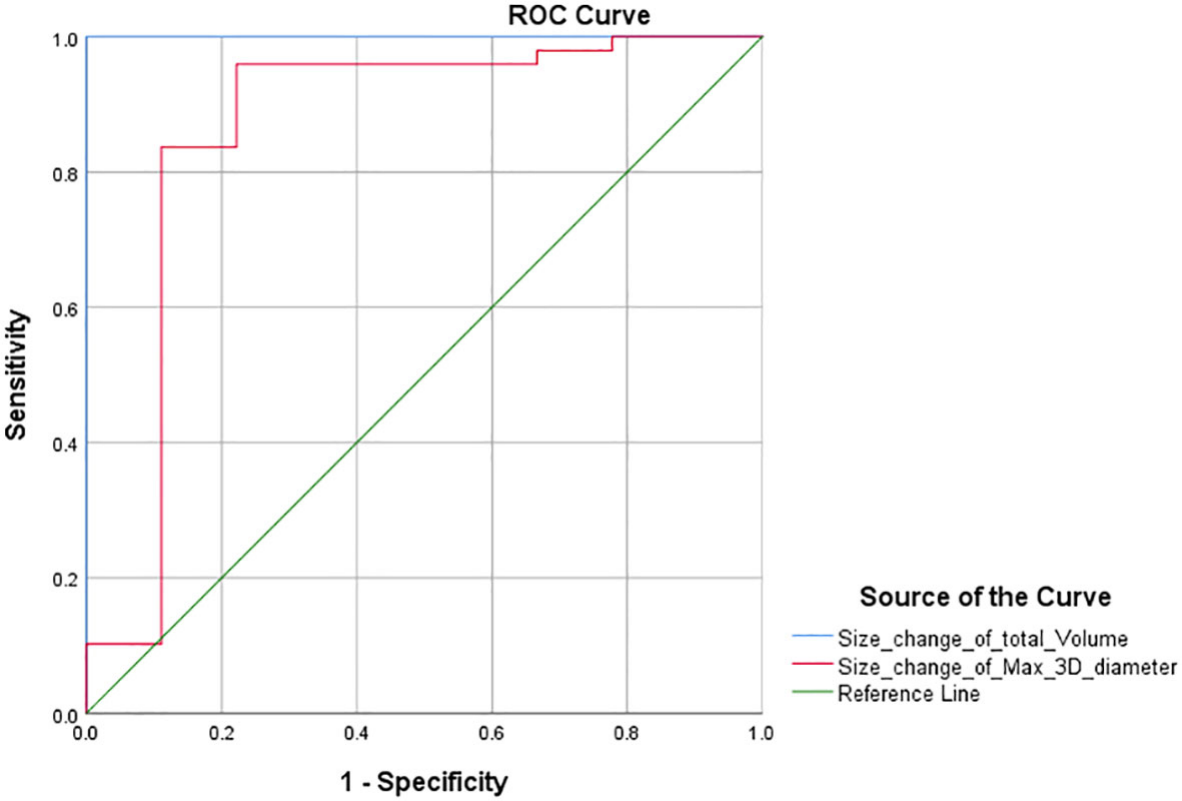
ROC curve with PR threshold as variable.

**Figure 4:**
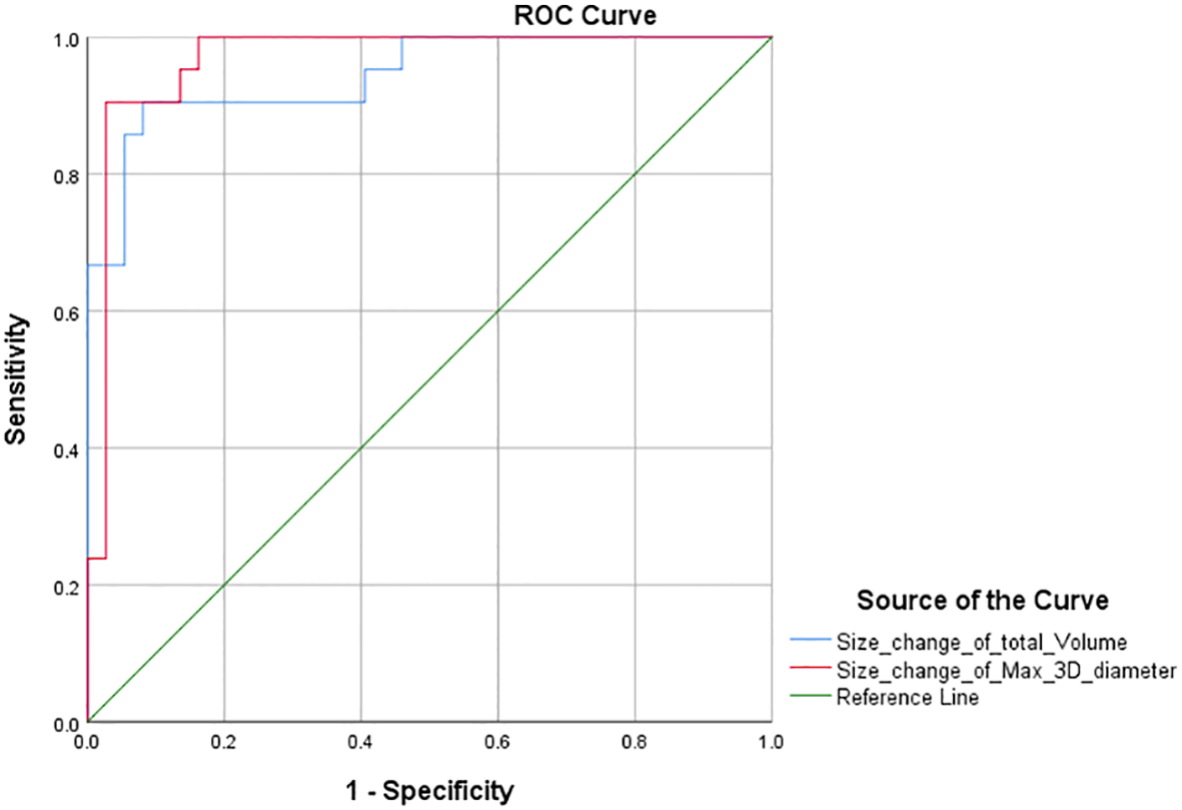
ROC curve with PD threshold as variable.

Furthermore, according to the PR threshold (−30%) of RECIST 1.1, we drew the ROC curve and obtained the AUC values for total volume and max. 3D diameter, which were 1 and 0.862 (**Figure 3**). We found that the maximum value of sensitivity plus specificity was 2. This corresponds to a volume size change of −64.9% and can be used as the new PR threshold for total volume. Similarly, according to the PD threshold of RECIST 1.1, the AUC values for total volume and max. 3D diameter were 0.945 and 0.968 (**Figure 4**) from the ROC curve. The maximum value of sensitivity plus specificity was 1.776. Its corresponding volumetric size change is 21.4%, which can be used as the new PD threshold for total volume.

## Discussion

In this retrospective study of patients with intracranial malignancies after immunotherapy, using a semi-automated segmentation technique, we found that, compared with RECIST 1.1, volumetric assessment and max. 3D diameter assessment had consistent efficacy in assessing immunotherapy response (P <0.05; Kappa >0.75; AUC >085). Besides, our initial studies found that when the volumetric threshold of PR is −64.9% and the volumetric threshold of PD is 21.4%, the sensitivity and specificity are both the highest. At this point, the assessment efficacy of the immunotherapy response is equal to −30% and +20% of RECIST 1.1.

There were relatively few existing studies about the assessment of radiological response in the nervous system after immunotherapy recently (9, 23–25). Therefore, in this study, we investigated the radiologic response of intracranial malignancies after immunotherapy. According to the iRANO guidelines, the gold standard for determining recurrence or progression of intracranial malignancies is a biopsy or pathological section (2). However, currently, biopsies often obtain only very small tissue aliquots, which may lead to sampling artifacts. On the other hand, in clinical practice, many patients may undergo surgery before immunotherapy when the primary malignancy is found, such as high-grade glioma. As a result, the immunotherapy response of intracranial malignancies would be based on follow-up MR examinations rather than biopsies or pathological sections. Malignancies such as brain metastases, for which the patients did not easily receive surgery, were also assessed by follow-up MR examinations. Although initial studies had been conducted to monitor tumor response to radiotherapy or chemotherapy by assessing metabolic or functional parameters, such as using perfusion MRI or dynamic computed tomography (CT), size change of target lesions remains the most widely used parameter to monitor therapy response (2, 26). For size changes of target lesions, the iRANO working group stated that RECIST 1.1 criteria were the recommended radiological assessment criteria of immunotherapy response (2, 6). Therefore, based on all the above, RECIST 1.1 was used as the gold standard in this study.

**Figure 5:**
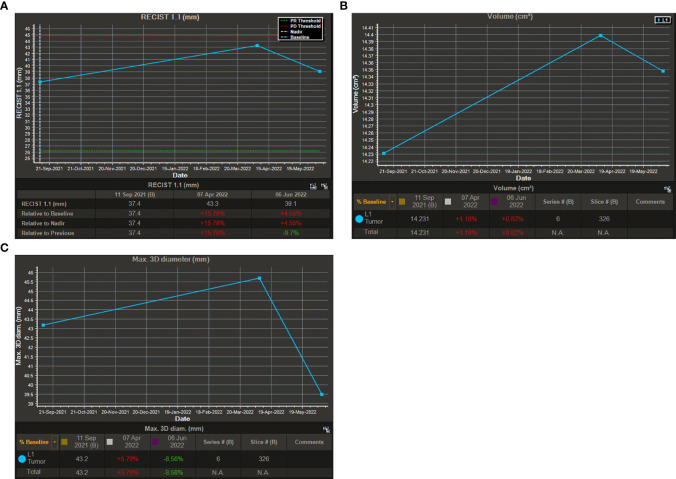
Visual charts of the semi-automatic segmentation technique. **(A)**: Visual chart of RECIST 1.1. **(B)**: Visual chart of total volume. **(C)**: Visual chart of max. 3D diameter.

Based on the optimized semi-automatic segmentation technology, one-stop intelligent identification and quantitative analysis of various tumors can be performed. It combines deep learning techniques to segment the boundaries of tumors accurately in a short time. The results support a variety of assessment criteria and can be output in a visual chart (**Figure 5**). Now, according to iRANO guidelines, one of the reasons why volumetric measurement remains controversial is that the technology increases costs and is not available in some centers. In this study, we performed a quantitative analysis of 78 MR examinations, including 20 MR examinations as a baseline and 58 longitudinal follow-up MR images as target assessments. The results showed that the time cost for quantitatively measuring the lesions on CE-MRI images is shorter (428.83 ± 88.47 s), and then we could obtain the results of RECIST 1.1, total volume, and max. 3D diameter simultaneously. According to our experiences, the semi-automatic segmentation time is equal to the time of post-processing in a brain CTA, so we consider that it can be practiced in clinical work.

The RECIST 1.1 value is calculated as the sum of the longest axial diameter of the target lesions in intracranial malignancies. Because tumor growth is multidirectional and irregular, the axial size change does not represent all directional changes (15). This means that for irregular tumors, RECIST 1.1 may underestimate or overestimate the real tumor size. For assessing linear changes in maximum lesion diameter, the RECIST 1.0 criteria were originally established using a theoretical model of a solid tumor. RECIST 1.1 is an update of RECIST 1.0 for the entire population of patients with solid tumors. In actual clinical practice, accurate volumetric measurement shows certain advantages because of the varying tumor morphology. The RANO working group considered that the necessity for volumetric measurements would be confirmed with increasing clinical knowledge reserves and research on volumetric response assessment and reporting (2).

The chi-square test of the volumetric response assessment and the RECIST 1.1 response assessment showed that the volumetric assessment had a relatively high consistency efficiency in patients with intracranial malignancies undergoing immunotherapy (**Table 5**). Its corresponding P-value was zero, which was less than 0.05, indicating that there was a statistical significance. Besides, the Kappa value was 0.742. The above data showed concordance and a strong correlation between RECIST 1.1 and total volume. In addition, according to the ROC curve of PR threshold (−30%), the AUC value of total volume was 1 (**Figure 4**), and according to the ROC curve of the PD threshold (20%), the AUC value of total volume was 0.945, both of which reflect good diagnostic efficacy (**Figure 5**). Based on the above data, we consider that volume assessment using semi-automated segmentation is feasible for immunotherapy response assessment in intracranial malignancies and can be used as an updated method for RECIST 1.1. This is consistent with iRANO criteria, indicating that it would be encouraged as a secondary end point in the future when feasible.

Similarly, the sum of the longest axial diameters cannot represent the changes in all dimensions, while the max. 3D diameter based on semi-automatic segmentation techniques is intelligently identified in any dimensions. Using a unidimensional threshold (**Table 2**), we performed a chi-square test according to the response assessment results based on the sum of max. 3D diameter (**Table 6**, P <0; 0.05 Kappa >0.75). The above data showed concordance and a strong correlation between RECIST 1.1 and max. 3D diameter. According to the ROC curve (**Figures 3**, **4**), the AUC of the size change in the max. 3D diameter was 0.862 for the PR threshold and 0.968 for the PD threshold, which indicated that the max. 3D diameter assessment had better diagnostic efficiency. Therefore, it is feasible to consider max. 3D diameter as another complementary parameter for immunotherapy response assessment.

If lesions grew proportionally and uniformly across all dimensions, according to the threshold for RECIST 1.1, a spherical volumetric threshold calculated by formula 4πr^2^/3 could be used as a reliable threshold for response assessment (**Table 3**). However, existing real-world data are insufficient to demonstrate the universality of volumetric response criteria in the population of patients with intracranial malignancies. At present, there are few studies focusing on volume response criteria, such as the optimization of volume threshold for liver metastases by Winter (10). While other studies on semi-automatic volumetric measurement mostly concern the comparison of repeatability and variability, there is no literature focusing on the optimization of volumetric threshold in intracranial malignancies (7).

Therefore, the volumetric threshold in different diseases needs more in-depth research. Our data showed that the PR threshold of volumetric change was −64.9%, which had a sensitivity of 100% and a specificity of 100% for the detection of target lesions with or without partial response. This was almost consistent with the conventional PR threshold of −65%. At the same time, our analysis showed that the volumetric threshold of PD was 21.4%, which was significantly different from the conventional threshold of 73%. The sensitivity and specificity of its detection of target lesions’ progression were 85.7% and 91.9%, respectively, which indicated that the accuracy of the current predicted PD threshold needs to be improved with more cases in the future.

Finally, due to the relatively small number of patients receiving immunotherapy for intracranial malignancies, it is better to enroll more patients in the future, as this is a meaningful and feasible study. Although MR images were obtained from different scanner manufacturers and there were inevitable differences between MR protocols, all MR scans met the minimum requirements for proper assessment according to RECIST 1.1; therefore, we think this did not significantly affect the results.

## Conclusion

In conclusion, based on the semi-automatic segmentation technique, we found that volumetric assessment and max. 3D diameter assessment was reliable and consistent with RECIST 1.1 in response assessment of patients with intracranial malignancies undergoing immunotherapy. Total volume and max. 3D diameter can be used as complementary methods for RECIST 1.1 to assist neurosurgeons in the multi-aspect assessment of immunotherapy responses. Meanwhile, the initial analysis showed that when the volumetric threshold of PR was −64.9% and the volumetric threshold of PD was 21%, the sensitivity and specificity were the highest. The efficacy of volumetric thresholds for immunotherapy response assessment was equal to −30% and +20% of RECIST 1.1. This study will be useful for guiding further treatments for patients.

## Data availability statement

The original contributions presented in the study are included in the article/supplementary material. Further inquiries can be directed to the corresponding author.

## Ethics statement

Ethical review and approval were not required for the study on human participants in accordance with the local legislation and institutional requirements. Written informed consent for participation was not required for this study in accordance with the national legislation and the institutional requirements.

## Author contributions

JT and CL provided the draft and performed the statistical analysis. JT, CL, ZL, HW, and YL collected the data. JT, MW, and CL evaluated the literature. JT wrote the first draft of the manuscript with support from other authors. RX, MW, SL, and CL revised the review. RX, YM, and MW contributed to the review. All authors contributed to the article and approved the submitted version.
